# Dysbiosis of fecal microbiota in cats with naturally occurring and experimentally induced *Tritrichomonas foetus* infection

**DOI:** 10.1371/journal.pone.0246957

**Published:** 2021-02-19

**Authors:** Metzere Bierlein, Barry A. Hedgespeth, M. Andrea Azcarate-Peril, Stephen H. Stauffer, Jody L. Gookin

**Affiliations:** 1 Department of Clinical Sciences, College of Veterinary Medicine and Comparative Medicine Institute, North Carolina State University, Raleigh, North Carolina, United States of America; 2 Division of Gastroenterology and Hepatology, Department of Medicine, UNC Microbiome Core, Center for Gastrointestinal Biology and Disease, School of Medicine, University of North Carolina, Chapel Hill, North Carolina, United States of America; University of Illinois, UNITED STATES

## Abstract

The protozoal pathogen *Tritrichomonas foetus* infects the colon of domestic cats and is a major cause of chronic colitis and diarrhea. Treatment failure is common, but antibiotics may improve clinical signs in a subset of cats, leading researchers to question involvement of the colonic microbiota in disease pathogenesis. Studies performed in women with venereal *Trichomonas vaginalis* infections have revealed that dysbiosis of host microbiota contributes to pathogenicity with similar findings also found in mice with intestinal *Tritrichomonas musculis* The aim of this study was to characterize differences in the fecal microbiota of cats with and without naturally occurring *T*. *foetus* infection and in a group of kittens prior to and after experimentally induced infection. Archived fecal DNA from cats undergoing testing for *T*. *foetus* infection (n = 89) and experimentally infected kittens (n = 4; at pre-, 2 weeks, and 9 weeks post-infection) were analyzed by sequencing of 16S rRNA genes. Amongst the naturally infected population, the genera *Megamonas* and *Helicobacter* were significantly increased in prevalence and abundance in cats testing positive for *T*. *foetus* infection. In the group of four experimentally infected kittens, fecal samples post-infection had significantly lower abundance of genus *Dialister* and *Megamonas* and greater abundance of the class *Betaproteobacteria* and family *Succinivibrionaceae*. We hypothesize that *T*. *foetus* promotes dysbiosis by competition for fermentable substrates used by these bacteria and that metabolic byproducts may contribute to the pathogenesis of colonic inflammation and diarrhea. Future studies are warranted for the measurement of fecal concentrations of microbial and protozoal metabolites in cats with *T*. *foetus* infection for the identification of potential therapeutic targets.

## Introduction

*Tritrichomonas foetus* is a common cause of chronic colitis and diarrhea in domestic cats worldwide [1–6]. Infections caused by this protozoan are notoriously difficult to treat as many isolates are resistant to current therapies and relapse is common [7–9]. Trichomonads are obligate parasites or commensals of anaerobic mucosa-lined body cavities such as the gastrointestinal and reproductive tract. These protozoa cause disease by a variety of mechanisms including disruption of the mucosal microenvironment [10, 11], adhesion and degradation of the mucosal barrier [12], induction of epithelial apoptosis [13, 14], and evasion of the host immune system [15, 16]. In cats, *T*. *foetus* infection is transmitted via the fecal-oral route and restricted in final residence to the colon where the organisms live intermixed with colonic bacteria in close proximity to the mucosa [2, 17]. Presence of the infection results in lymphocytic, plasmacytic, and neutrophilic colitis and chronic, oftentimes lifelong, diarrhea [18].

Studies in women with the venereal protozoan *Trichomonas vaginalis* pinpoint dysbiotic vaginal microbiota as mediating not only pathogenic effects of infection but also predisposition to infection [19–22]. The dysbiotic bacteria serve as pathobionts that enhance pathogenicity by promoting adhesion of *T*. *vaginalis* to cervical cells [21] and increasing paracellular permeability [20]. In laboratory mice, colonization of the intestine by commensal *Tritrichomonas musculis* is influenced by competitive and cooperative relationships with members of the intestinal microbiota for metabolic byproducts [23]. Trichomonads are reliant on acquisition of micronutrients from the host environment and may also prey upon host bacteria via phagocytosis [24]. Currently, no studies have investigated the impact of *T*. *foetus* infection on the colonic microbiota of cats. This relationship is particularly interesting as treatment with antibiotics may improve clinical signs of diarrhea in some cats with *T*. *foetus* infection while precipitating a relapse of diarrhea in others [1, 3, 9].

Our objective in this study was to characterize the impact of naturally occurring and experimentally introduced *T*. *foetus* infection on the colonic microbiota of cats using Illumina-based 16S rRNA gene sequencing of archived fecal DNA. We hypothesized that cats with *T*. *foetus* infection would have a dysbiosis of specific members of the fecal microbiota whose characteristics could provide insights into disease pathogenesis and targets for treatment.

## Methods

### Fecal DNA samples

#### Cats having PCR testing for diagnosis of naturally occurring *T*. *foetus* infection

Archived DNA was identified for fecal samples submitted for *T*. *foetus* PCR testing to the North Carolina State University Intestinal Pathogens Research Laboratory between the years of 2012 to 2017. DNA extractions were performed using a Zymo Fecal/Soil DNA extraction kit (Zymo Research, Irvine CA) as previously described [25]. All DNA samples were stored at -20°C prior to use in the current study. A complete description of the parent population from which these samples were selected was recently published [26]. Inclusion criteria for this study were samples originally accompanied by a submission form in which the treatment history, or indication of lack of treatment, was provided. Additional patient information extracted from the submission forms included breed, age, life stage [27], sex, method of fecal collection (voided, loop, colonic flush), and indication for and results of *T*. *foetus* PCR testing. As previously described [5], loop collections are performed by inserting a fecal loop device through the anus into the colon to collect a fecal sample. A colonic flush is performed by inserting an 8 to 10 French red rubber catheter through the anus and instilling saline into the proximal colon to create a fecal slurry. The slurry material is collected and the sediment is used as the fecal sample. Samples were excluded from the study if no treatment history was provided or if the treatment history reported any prior administration of antimicrobial or anthelminthic drugs.

#### Kittens experimentally infected with feline *T*. *foetus*

Archived DNA was identified for fecal samples collected from kittens prior to and after experimental infection with *T*. *foetus* in 2006 [28]. DNA extractions were performed using a QIAamp DNA Stool minikit (Qiagen) with minor modifications as previously described [29]. All DNA samples were stored at -20°C prior to use in the current study. For the experimental infection, four specific pathogen free 7-week old sexually intact male (n = 2) and female (n = 2) domestic shorthair kittens were purchased from the same vendor (Liberty Laboratories, Liberty, NY). Each kitten was housed in a separate cage in the same laboratory animal resources facility and received the same dry food ad libitum (Hill’s Science Diet Feline Growth) throughout the study. After an acclimation period of seven weeks, and at an age of 14 weeks, kittens were infected with a single isolate of *T*. *foetus* that was cultivated from feces of a cat with diarrhea [28]. Each kitten was inoculated with 2 x 10^6^ *T*. *foetus* by orogastric intubation. Included in this study are DNA samples extracted from feces collected from each kitten using a fecal loop prior to infection and at 2 and 9 weeks post-infection. The study was approved by the North Carolina State University Institutional Animal Care and Use Committee #03-095B. Due to inability to later eradicate the induced infection, cats were euthanized by IV injection of pentobarbital at the conclusion of the original study.

### Bacterial 16S rRNA gene amplification and sequencing

Extracted DNA was quantified via PicoGreen analysis and used for bacterial 16S rRNA gene amplicon sequencing. Total DNA (12.5 ng) was amplified using universal primers targeting the V4 region of the bacterial 16S rRNA gene [30]. The sequences of the primers were: 515F - 5′ TCGTCGGCAGCGTCA GATGTGTATAAGAGACAGGTGCCAGCMGCCGCGGTAA 3′ and 806R - 5′GTCTCGTGGGCTCGGAGATGT GTATAAGAGACAGGGACTACHVGGGTWTCTAAT 3′. Overhang adapters were appended to the 5′ end of each primer sequence for compatibility with the Illumina sequencing platform. Master mixes contained 12.5 ng of total DNA, 0.2 μM of each primer and 2× KAPA HiFi HotStart ReadyMix (KAPA Biosystems, Wilmington, MA). The thermal profile for the amplification of each sample had an initial denaturing step at 95°C for 3 minutes, followed by 25 cycles of denaturing of 95°C for 30 seconds, annealing at 55°C for 30 seconds for 16S rRNA and a 30 second extension at 72°C, a 5 minute extension at 72°C and a final hold at 4°C. Each 16S amplicon was purified using the AMPure XP reagent (Beckman Coulter, Indianapolis, IN). In the next step each sample was amplified using a limited cycle PCR program, adding Illumina sequencing adapters and dual index barcodes (index 1(i7) and index 2(i5)) (Illumina, San Diego, CA) to the amplicon target. The thermal profile for the amplification of each sample had an initial denaturing step at 95°C for 3 minutes, followed by a denaturing cycle of 95°C for 30 seconds, annealing at 55°C for 30 seconds and a 30 second extension at 72°C (8 cycles), a 5 minute extension at 72°C and a final hold at 4°C. The final libraries were again purified using the AMPure XP reagent (Beckman Coulter), quantified with Quant-iT™ PicoGreen® dsDNA Reagent (Molecular Probes, Thermo Fisher Scientific, Waltham, MA), and normalized prior to equimolar pooling. The DNA library pool was then denatured with NaOH, diluted with hybridization buffer and heat denatured before loading on the MiSeq reagent cartridge MiSeq instrument (Illumina). Automated cluster generation and paired–end sequencing with dual reads were performed according to the manufacturer’s instructions.

### Sequencing data analysis

Sequencing output from the Illumina MiSeq platform were converted to fastq format and demultiplexed using Illumina Bcl2Fastq 2.18.0.12. The resulting paired-end reads were processed using QIIME 2 [31] 2018.11. Index and linker primer sequences were trimmed using the QIIME 2 invocation of cutadapt. The resulting paired-end reads were processed with DADA2 through QIIME 2 including merging paired ends, quality filtering, error correction, and chimera detection. Amplicon sequencing units from DADA2 were assigned taxonomic identifiers with respect to Green Genes release 13_08.

Alpha diversity estimates were calculated within QIIME 2 using Evenness (Shannon) index and observed species number metrics at a rarefaction depth of 5,000 reads. Pairwise significance was tested using a Kruskal-Wallis ANOVA with Benjamini-Hochberg corrected q-values calculated as implemented in QIIME 2. Beta diversity estimates were calculated within QIIME 2 using weighted and unweighted Unifrac distances as well as Bray-Curtis dissimilarity between samples at a subsampling depth of 5,000 reads. Results were summarized, visualized through principal coordinate analysis in Emperor, and significance was estimated by PERMANOVA with Benjamini-Hochberg corrected q-values calculated as implemented in QIIME 2. Differentially abundant microbial taxa were identified by Analysis of Composition of Microbiomes (ANCOM) in QIIME 2. Relative abundance data were normalized by sample library size and taxa were removed if they were present in less than 10% of all samples or if lower than 0.01% average abundance [32]. Differences in abundance were tested for significance using a Kruskal-Wallis One-Way Analysis of Variance on Ranks. When significant, pairwise comparisons between groups were performed using a Dunn’s test. Generated p-values were corrected for multiple testing using the Benjamini-Hochberg procedure [33] at a false discovery rate of 0.15. For selected taxa, odds ratio and 95% confidence intervals for association with infection status were calculated using a Chi-square Test with Yates Continuity Correction. Statistical examination of abundance data were conducted using Systat Software (SigmaPlot 12.0, San Jose, CA). Results were represented graphically using GraphPad Software (Prism version 7.03, San Diego, CA).

## Results

### Cats with naturally occurring *T*. *foetus* infection

From a total of 1,717 archived DNA samples extracted from feces submitted for *T*. *foetus* PCR testing, 89 samples met criteria for inclusion in the study. Sixteen breeds were represented including domestic (46 cats), Bengal (11), Abyssinian (7), and Ragdoll (6) most commonly. Mean and median ages of cats were 3.7 and 1.7 years respectively (range 3 months– 16 years). The majority of cats were classified by life stage as junior (28 cats), followed by kitten (20 cats), prime (19 cats), mature (14 cats), senior (2 cats), geriatric (1 cat), or unknown age (2 cats). Sexes included male castrated (35 cats), female spayed (24 cats), female intact (16 cats), and male intact (14 cats). Test results for *T*. *foetus* infection, clinical signs, method of fecal sample collection, and treatment history of cats from which DNA samples were included in the study are shown in Table 1.

**Table 1:** Clinical data pertaining to 89 cats that underwent PCR testing for *T*. *foetus* infection and had archived fecal DNA used in this study.

Clinical Variable	PCR test result for *T*. *foetus* infection No. (%)	
	Positive	Negative
PCR test results	17 (19)	72 (81)
Clinical signs		
Diarrhea	12 (13)	54 (61)
No diarrhea	5 (6)	16 (18)
Not reported	0 (0)	2(2)
Method of fecal collection		
Voided	8 (9)	33 (37)
Fecal loop	4 (4)	16 (18)
Colon flush	4(4)	21 (24)
Not reported	1 (1)	2 (2)
Treatment history†		
Prescription diet	5 (6)	5 (6)
Probiotic	3 (3)	11 (12)
Steroid	1 (1)	10 (11)
Supplement	0 (0)	4 (4)
No treatment	9 (10)	49 (55)

Specialty diets included unspecified (6 cats), Hills Prescription Diet I/D (2), Purina EN (1), or a duck-based diet (1). Probiotics used included Purina Fortiflora (10), unspecified (3), or NutraMax Proviable (1). Specific steroids used were prednisolone (10) or Depomedrol (1). Supplements included one each of Diagel, Benefiber, or Clay.

†Some cats received more than 1 listed treatment.

### Major phyla in fecal microbiota of cats undergoing testing for *T*. *foetus* infection include Firmicutes, Bacteroidetes, Proteobacteria, Fusobacteria, and Actinobacteria

**Fig 1:**
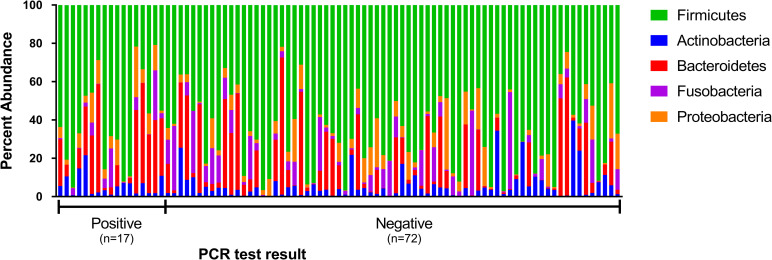
Percent abundance of the major phyla observed in feces from 89 individual cats undergoing testing for *T*. *foetus* infection. Each sample is designated as having been obtained from a cat testing negative versus positive for *T*. *foetus* infection as determined by results of fecal PCR testing.

The sequence analysis of all 89 cats yielded 9,691,382 quality sequences (mean ± SD = 108,892 ± 21,089). The major phyla represented in the fecal microbiota of cats tested for *T*. *foetus* infection were Firmicutes, Bacteroidetes, Proteobacteria, Fusobacteria, and Actinobacteria (Fig 1).

**Fig 2:**
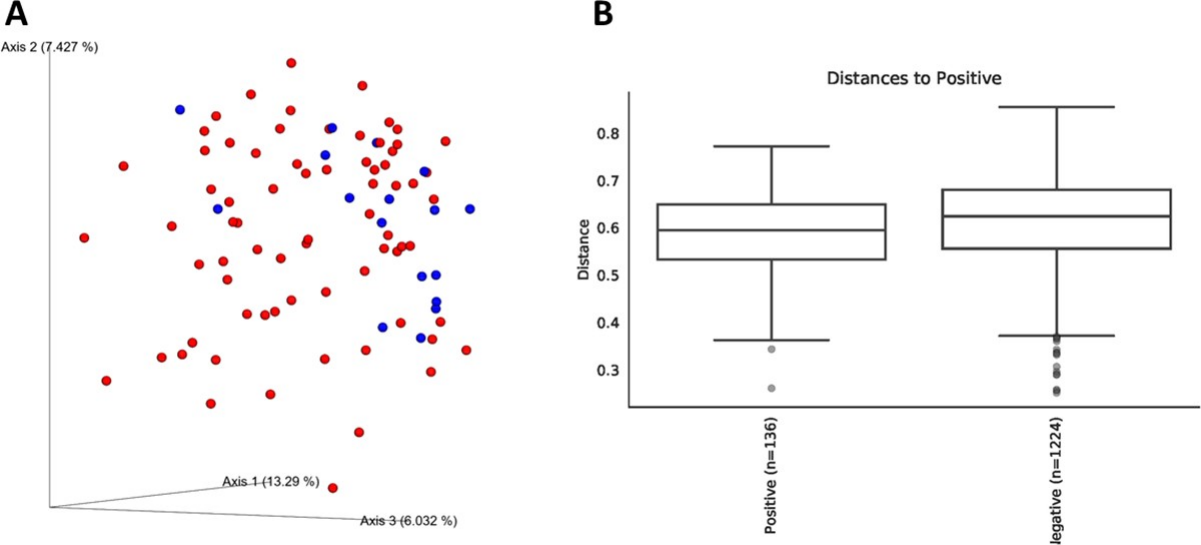
Beta diversity of microbiota from 89 cats tested for naturally occurring infection with *T*. *foetus*. Panel A: Bray-Curtis principal coordinates analysis plot showing clustering of microbial communities from feces of cats testing positive (blue circles) versus negative (red circles) for *T*. *foetus* infection. Axis 1, 13.29%; Axis 2, 7.427%; Axis 3, 6.032%. Panel B: Unweighted Unifrac distances between microbial communities from feces testing positive versus negative for *T*. *foetus* infection. PERMANOVA q = 0.033.

There were no significant differences in alpha diversity measures of evenness (Shannon), diversity (Faith), or observed number of taxa between cats testing positive versus negative for *T*. *foetus* infection. Both weighted and unweighted measures of beta diversity identified small but significant differences in phylogenetic composition of the microbiota between cats testing positive versus negative for naturally occurring *T*. *foetus* infection (PERMANOVA, q < 0.05) (Fig 2).

### Cats testing positive for *T*. *foetus* infection have more frequent membership and higher abundance of genus *Megamonas* and *Helicobacter*

Abundance of specific taxa observed in the feces of cats testing positive versus negative for naturally occurring *T*. *foetus* infection are shown in Table 2.

**Table 2:** Relative abundance of genus level taxa (percent of total sequences) in the fecal microbiota of cats undergoing PCR testing for naturally occurring *T*. *foetus* infection.

Phylum	Family	Genus	Median % abundance [range min to max]		Kruskal-Wallis ANOVA p-value
			PCR Positive (n = 17 cats)	PCR Negative (n = 72 cats)	
*Actinobacteria*			5.19 [0.78–21.59]	3.63 [0.04–39.61]	0.484
	*Bifidobacteriaceae*	*Bifidobacterium*	0.03 [0.0–5.79]	0.02 [0.0–31.05]	0.688
	*Coriobacteriaceae*	*Uncl*. *Coriobacteriaceae*	0.09 [0.0–0.75]	0.04 [0.0–1.21]	0.342
		*Adlercreutzia*	0.05 [0.0–0.12]	0.02 [0.0–0.21]	0.141
		*Collinsella*	3.05 [0.24–18.62]	2. 80 [0.0–12.24]	0.262
		*Slackia*	0.05 [0.0–0.35]	0.08 [0.0–1.56]	0.431
*Bacteroidetes*			24.40 [0.05–56.30]	4.48 [0.01–71.26]	0.115
	*Bacteroidaceae*	*Bacteroides*	0.63 [0.0–29.94]	0.96 [0.0–43.68]	0.774
	*Prevotellaceae*	*Prevotella*	9.71 [0.02–50.82]	1.27 [0.0–47.79]	0.075
	*[Paraprevotellaceae]*	*[Prevotella]*	0.10 [0.0–7.75]	0.02 [0.0–23.98]	0.885
*Firmicutes*			63.66 [20.84–95.03]	65.32 [21.71–97.00]	0.342
	*Enterococcaceae*	*Enterococcus*	0.06 [0.0–12.57]	0.08 [0.0–47.67]	0.7
	*Turicibacteraceae*	*Turicibacter*	0.0 [0.0–3.51]	0.01 [0.0–48.24]	0.142
	*Streptococcaceae*	*Streptococcus*	0.01 [0.0–10.00]	0.02 [0.0–49.65]	0.179
	*Uncl*. *Clostridiales*	*Uncl*. *Clostridiales*	0.07 [0.0–0.62]	0.21 [0.0–5.64]	0.071
	*Clostridiaceae*	*Clostridium*	5.55 [0.86–57.11]	7.26 [0.04–59.92]	0.888
	*Lachnospiraceae*	*Uncl*. *Lachnospiraceae*	3.58 [0.93–40.76]	3.59 [0.01–25.73]	0.822
		*Blautia*	5.50 [2.01–27.11]	8.11 [0.0–38.68]	0.806
		*Clostridium*	0.02 [0.0–8.11]	0.04 [0.0–5.48]	0.707
		*Coprococcus*	0.13 [0.0–2.24]	0.19 [0.0–7.99]	0.378
		*Dorea*	0.75 [0.0–3.31]	0.61 [0.0–9.00]	0.896
		*Roseburia*	0.29 [0.0–2.92]	0.06 [0.0–18.42]	0.772
		*[Ruminococcus]*	1.52 [0.19–9.28]	1.40 [0.0–9.57]	0.719
	*Peptococcaceae*	*Peptococcus*	0.43 [0.0–4.29]	0.93 [0.0–19.38]	0.241
	*Peptostreptococcaceae*	*Uncl*. *Peptostreptococcaceae*	0.52 [0.0–15.03]	0.37 [0.0–83.14]	0.637
	*Ruminococcaceae*	*Uncl*. *Ruminococcaceae*	0.70 [0.0–2.28]	1.05 [0.0–12.31]	0.407
		*Faecalibacterium*	0.13 [0.0–2.43]	0.15 [0.0–9.64]	0.851
		*Oscillospira*	0.13 [0.0–1.87]	0.28 [0.0–1.90]	0.43
		*Ruminococcus*	0.03 [0.0–0.27]	0.03 [0.0–6.41]	0.975
	*Veillonellaceae*	*Dialister*	0.16 [0.0–3.03]	0.03 [0.0–4.11]	0.327
		*Megamonas*	1.52 [0.0–17.31]	0.03 [0.0–7.39]	0.001*** †
		*Megasphaera*	1.37 [0.0–14.27]	0.11 [0.0–15.54]	0.116
		*Phascolarctobacterium*	0.30 [0.0–3.15]	0.03 [0.0–7.32]	0.42
	*[Mogibacteriaceae]*	*Uncl*. *[Mogibacteriaceae]*	0.22 [0.0–6.54]	0.09 [0.0–2.55]	0.325
	*Erysipelotrichaceae*	*Bulleidia*	0.04 [0.0–4.07]	0.09 [0.0–9.59]	0.974
		*Catenibacterium*	0.44 [0.0–7.25]	0.09 [0.0–5.46]	0.121
		*[Eubacterium]*	0.83 [0.02–8.04]	0.60 [0.0–11.56]	0.855
*Fusobacteria*			0.59 [0.01–25.95]	2.08 [0.0–50.63]	0.251
	*Uncl*. *Fusobacteriaceae*	*Uncl*. *Fusobacteriaceae*	0.59 [0.0–24.24]	1.52 [0.0–28.00]	0.507
	*Fusobacteriaceae*	*Fusobacterium*	0.0 [0.0–0.91]	0.03 [0.0–6.99]	0.334
*Proteobacteria*	* *	* *	6.40 [0.33–26.37]	3.18 [0.0–37.26]	0.306
	*Alcaligenaceae*	*Sutterella*	0.34 [0.0–2.96]	0.59 [0.0–5.88]	0.389
	*Campylobacteraceae*	*Campylobacter*	0.06 [0.0–3.96]	0.01 [0.0–15.88]	0.050*
	*Succinivibrionaceae*	*Anaerobiospirillum*	1.62 [0.0–11.27]	0.01 [0.0–23.51]	0.046*
	*Enterobacteriaceae*	*Uncl*. *Enterobacteriaceae*	0.01 [0.0–2.34]	0.05 [0.0–22.12]	0.264
	*Helicobacteraceae*	*Helicobacter*	0.09 [0.0–5.13]	0.0 [0.0–5.25]	0.003**†

*p<0.05 and

***p<0.001.

†Benjamini-Hochberg corrected p-value <0.15.

**Fig 3:**
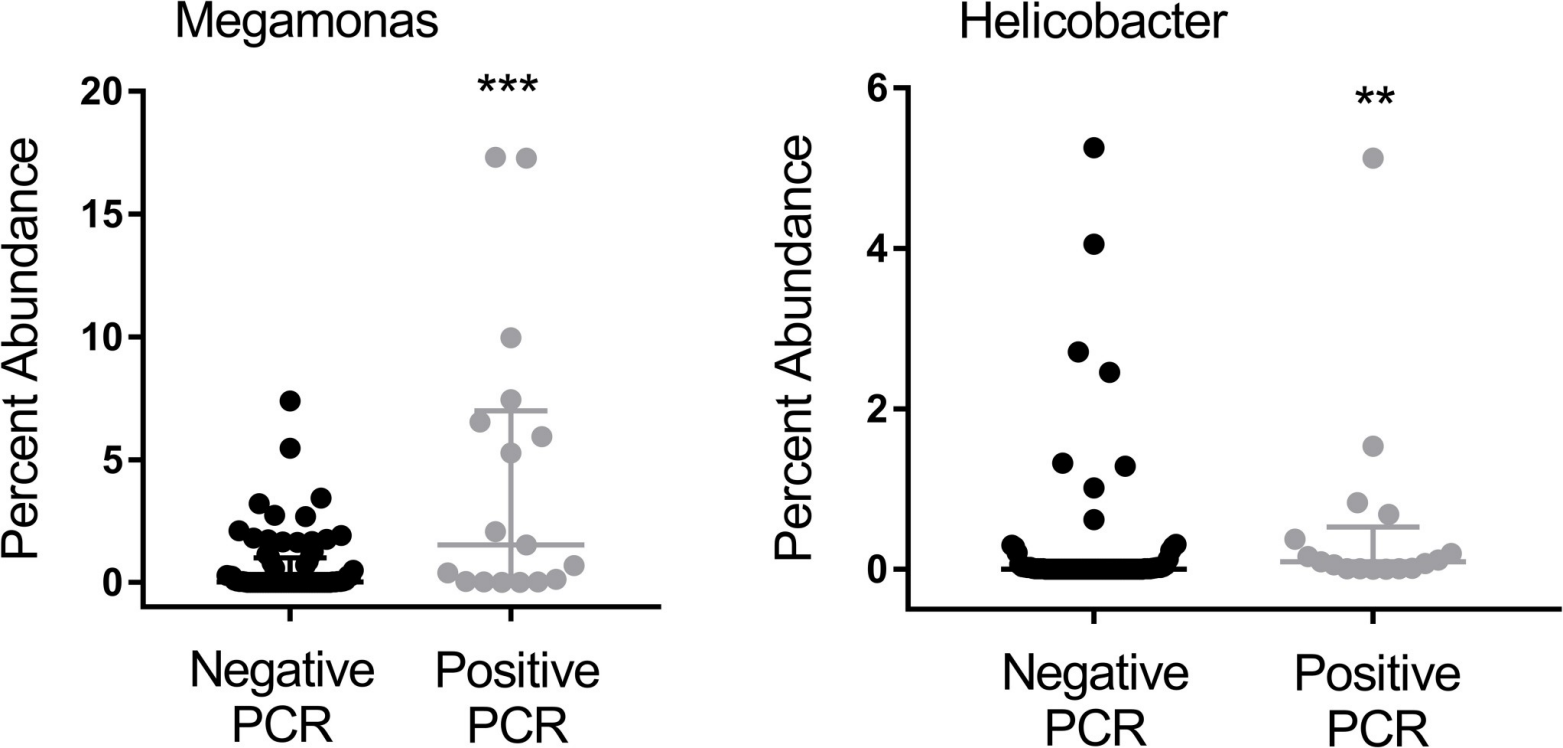
Percent abundance of *Megamonas* and *Helicobacter* in feces of cats testing PCR negative (n = 72 cats) or PCR positive (n = 17 cats) for naturally occurring *T*. *foetus* infection. Data points represent individual cats. Bars represent median and interquartile range. Mann-Whitney Rank Sum Test ***p = 0.001 (BH-corrected p = 0.043). **p = 0.003 (BH-corrected p = 0.06). BH = Benjamini-Hochberg.

After accounting for the impact of multiple statistical comparisons, cats testing positive for *T*. *foetus* infection had a significantly greater abundance of members of the genus *Megamonas* and *Helicobacter* compared to cats that tested negative for *T*. *foetus* infection (Fig 3).

Cats testing positive for *T*. *foetus* infection were 10.8 times more likely to have *Megamonas* (95%CI = 1.356 to 85.9, p = 0.016, Chi-square test) and 7.9 times more likely to have *Helicobacter* present in the microbial community (95%CI = 1.689 to 37.210, p = 0.007, Chi-square test) compared to cats testing negative for *T*. *foetus* infection.

### Method of fecal sample collection significantly influence the abundance of select taxa in feces of cats tested for *T*. *foetus* infection

**Fig 4:**
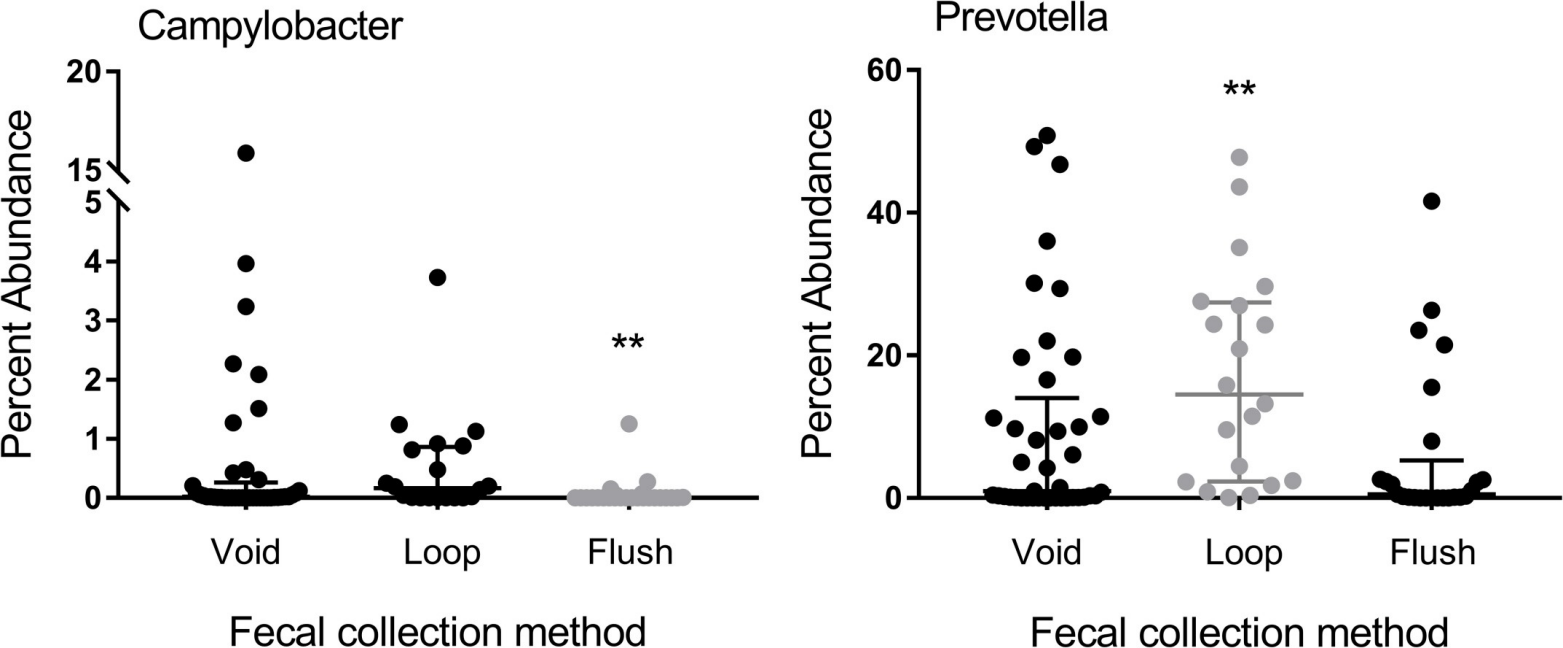
Percent abundance of *Campylobacter* and *Prevotella* in feces of cats having the sample collected after voiding (n = 41 cats), using a fecal loop (n = 20 cats), or by means of colonic flush (n = 25 cats). Data points represent individual cats. Bars represent median and interquartile range. Kruskal-Wallis One Way ANOVA on Ranks **p<0.01 (BH-corrected p = 0.129). BH = Benjamini- Hochberg.

When examining the impact of available metadata in the cats undergoing testing for *T*. *foetus* infection, no significant differences in alpha or beta diversity were observed based on life stage, breed, sex, method of fecal sample collection, clinical signs of diarrhea, or treatment history. However, method of fecal sample collection had a significant impact on relative abundance of members of the genus *Prevotella* and *Campylobacter* (Fig 4).

*Campylobacter* was less abundant in feces collected by colonic flush compared to samples collected after voiding or using a fecal loop. Fecal samples collected using a loop recovered a greater percent abundance of *Prevotella* compared to samples collected after voiding or by colonic flush.

### Cats with experimentally induced *T*. *foetus* infection

Following orogastric infection, all four specific pathogen free kittens were confirmed positive for *T*. *foetus* by means of fecal DNA PCR testing at both the 2 week and 9 week post-infection time points. None of the kittens developed clinical signs of diarrhea at any time point following infection.

The sequence analysis yielded 1,408,793 quality sequences for the 12 analyzed fecal samples (mean ± SD = 117,399 ± 26,660). Among the measures of alpha diversity, significant differences were observed between pre- and post-infection fecal samples in the evenness of species abundances (Shannon alpha diversity; Kruskal-Wallis q = 0.04) but not in the number of different taxa observed.

**Fig 5:**
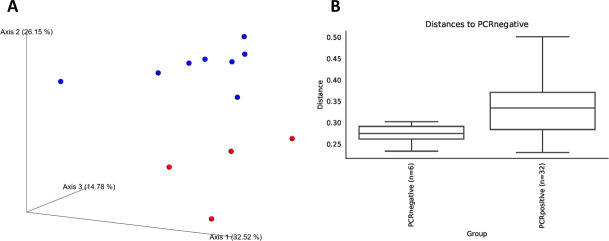
Bray-Curtis beta diversity of microbiota from 4 kittens prior to (PCR negative) and at 2 time points after (PCR positive) experimental infection with feline *T*. *foetus*. Panel A: Principal coordinates analysis plot showing clustering of microbial communities from feces of cats prior to (red circles) and after (blue circles) experimental *T*. *foetus* infection. Axis 1, 35.52%; Axis 2, 26.15%; Axis 3, 14.78%. Panel B: Weighted Unifrac distances between microbial communities from feces of cats prior to (PCR negative) and after (PCR positive) experimental *T*. *foetus* infection. Bray-Curtis PERMANOVA q = 0.003.

Weighted measures of beta diversity identified significant differences in phylogenetic composition of the fecal microbiota between pre- and post-infection samples from cats with experimentally induced *T*. *foetus* infection (PERMANOVA, q < 0.01) (Fig 5).

### Experimental infection with *T*. *foetus* shifts the abundance of specific taxa in the feline fecal microbiome

Abundance of specific taxa observed in the feces of kittens prior to and at two time points after undergoing experimental infection with *T*. *foetus* are shown in Table 3.

**Table 3:** Relative abundance of genus level taxa (percent of total sequences) in the fecal microbiota of 4 cats prior to and following experimentally induced *T*. *foetus* infection.

			Median % abundance [Range min to max]			Kruskal-Wallis ANOVA
Phylum	Family	Genus	Pre-infection	2 weeks post-infection	9 weeks post-infection	
						p-value
*Actinobacteria*			1.45 [0.25–4.72]	0.54 [0.18–1.21]	0.39 [0.01–2.84]	0.706
	*Bifidobacteriaceae*	*Bifidobacterium*	0.01 [0.0–0.03]	0.00 [0.0–0.0]	0.00 [0.0–0.01]	0.136
	*Coriobacteriaceae*	*Adlercreutzia*	0.05 [0.01–0.1]	0.04 [0.01–0.11]	0.06 [0.0–0.12]	1.000
		*Collinsella*	1.33 [0.23–4.65]	0.48 [0.13–1.03]	0.33 [0.01–2.55]	0.706
*Bacteroidetes*			60.16 [51.79–61.82]	59.53 [53.31–64.53]	56.81 [50.80–66.33]	0.815
	*Bacteroidaceae*	*Bacteroides*	10.45 [9.64–16.54]	16.21 [15.17–17.05]	16.23 [14.61–23.30]	0.136
	*Porphyromonadaceae*	*Parabacteroides*	1.31 [0.52–2.23]	2.14 [2.02–2.45]	1.60 [1.38–2.17]	0.197
	*Prevotellaceae*	*Prevotella*	29.69 [25.78–33.00]	21.08 [18.70–27.07]	25.26 [9.18–27.40]	0.044*
	*Rikenellaceae*	*Uncl*. *Rikenellaceae*	0.00 [0.0–0.03]	0.08 [0.0–0.18]	0.06 [0.0–0.14]	0.173
		*Alistipes*	0.18 [0.01-.33]	0.36 [0.08–0.95]	0.09 [0.05–0.92]	0.540
	*S24-7*	*Uncl*. *S24-7*	0.77 [0.61–0.94]	1.72 [0.88–2.75]	0.90 [0.40–1.01]	0.074
	*[Odoribacteraceae]*	*Odoribacter*	0.43 [0.16–1.04]	0.77 [0.62–0.95]	0.59 [0.22–1.08]	0.592
	*[Paraprevotellacea]*	*Paraprevotella*	0.07 [0.04–0.13]	0.10 [0.04–0.17]	0.11 [0.08–0.23]	0.397
		*[Prevotella]*	13.01 [9.36–21.52]	15.10 [13.51–19.13]	14.77 [9.37–19.92]	0.770
*Firmicutes*			28.96 [27.83–36.96]	22.60 [20.25–26.23]	22.01 [18.24–30.92]	0.086
	*Lactobacillaceae*	*Lactobacillus*	0.00 [0.0–0.01]	0.00 [0.0–0.01]	0.00 [0.0–0.03]	0.941
	*Leuconostocaceae*	*Leuconostoc*	0.00 [0.0–0.0]	0.00 [0.0–0.01]	0.00 [0.0–0.01]	0.630
	*Streptococcaceae*	*Lactococcus*	0.01 [0.0–0.02]	0.01 [0.0–0.02]	0.03 [0.01–0.03]	0.040*
		*Streptococcus*	0.00 [0.0–0.06]	0.00 [0.0–0.21]	0.01 [0.0–0.03]	0.968
	*Uncl*. *Clostridiales*	*Uncl*. *Clostridiales*	0.73 [0.60–1.35]	1.57 [0.57–1.98]	1.27 [0.71–2.06]	0.436
	*Clostridiaceae*	*Candidatus Arthromitus*	0.16 [0.0–0.46]	0.13 [0.01–0.65]	0.03 [0.01–0.13]	0.480
		*Clostridium*	0.36 [0.15–0.89]	0.26 [0.09–0.46]	0.16 [0.07–0.54]	0.452
	*Lachnospiraceae*	*Uncl*. *Lachnospiraceae*	1.94 [0.75–2.58]	2.77 [1.79–3.21]	2.42 [2.34–4.76]	0.252
		*Blautia*	0.39 [0.20–0.72]	0.34 [0.19–0.43]	0.35 [0.12–1.00]	0.913
		*Clostridium*	0.07 [0.0–0.15]	0.02 [0.02–0.03]	0.01 [0.0–0.04]	0.397
		*Coprococcus*	0.03 [0.0–0.03]	0.04 [0.01–0.04]	0.03 [0.01–0.19]	0.540
		*Dorea*	0.10 [0.04–0.32]	0.05 [0.01–0.12]	0.02 [0.01–0.08]	0.234
		*Lachnospira*	0.00 [0.0–0.16]	0.43 [0.0–0.69]	0.60 [0.07–1.11]	0.074
		*Roseburia*	0.54 [0.17–0.83]	0.36 [0.01–0.47]	0.77 [0.13–4.21]	0.592
		*[Ruminococcus]*	0.12 [0.09–0.27]	0.07 [0.06–0.14]	0.09 [0.07–0.45]	0.277
	*Peptococcaceae*	*Peptococcus*	0.16 [0.02–0.25]	0.08 [0.03–0.18]	0.07 [0.03–0.16]	0.706
	*Peptostreptococcaceae*	*Uncl*. *Peptostreptococcaceae*	0.69 [0.12–6.23]	0.59 [0.07–1.55]	0.08 [0.0–0.52]	0.252
	*Ruminococcaceae*	*Uncl*. *Ruminococcaceae*	1.75 [0.65–2.70]	1.47 [1.14–2.82]	1.28 [0.69–3.71]	0.968
		*Butyricicoccus*	0.01 [0.01–0.01]	0.01 [0.0–0.01]	0.01 [0.01–0.03]	0.592
		*Faecalibacterium*	2.68 [1.83–5.03]	3.36 [2.26–4.40]	2.87 [0.65–3.75]	0.840
		*Oscillospira*	1.14 [0.40–1.54]	1.03 [0.71–1.37]	0.94 [0.61–1.34]	0.941
		*Ruminococcus*	0.13 [0.02–0.21]	0.22 [0.19–0.27]	0.21 [0.16–0.47]	0.074
	*Veillonellaceae*	*Acidaminococcus*	0.75 [0.08–2.66]	0.33 [0.07–0.92]	0.09 [0.01–0.23]	0.145
		*Dialister*	10.54 [8.42–11.66]	6.17 [5.06–7.30]	7.09 [6.72–7.69]	0.002**†
		*Megamonas*	0.83 [0.72–2.14]	0.18 [0.18–0.20]	0.10 [0.04–0.46]	0.005**†
		*Megasphaera*	2.36 [1.49–3.65]	0.77 [0.47–1.92]	0.56 [0.26–0.68]	0.016*
		*Mitsuokella*	0.05 [0.0–0.82]	0.00 [0.0–0.15]	0.00 [0.0–0.01]	0.706
	*[Mogibacteriaceae]*	*Uncl*. *[Mogibacteriaceae]*	0.79 [0.24–1.17]	0.41 [0.15–0.62]	0.34 [0.14–0.83]	0.252
	*Erysipelotrichaceae*	*Uncl*. *Erysipelotrichaceae*	0.05 [0.0–0.09]	0.00 [0.0–0.0]	0.03 [0.0–0.14]	0.131
		*Allobaculum*	0.01 [0.0–0.03]	0.07 [0.0–0.25]	0.04 [0.02–0.18]	0.370
		*Bulleidia*	0.00 [0.0–0.10]	0.00 [0.0–0.19]	0.00 [0.0–0.49]	0.941
		*Catenibacterium*	0.41 [0.13–0.72]	0.46 [0.18–0.62]	0.38 [0.12–1.63]	0.968
		*Holdemania*	0.02 [0.0–0.02]	0.02 [0.02–0.04]	0.02 [0.01–0.94]	0.540
		*[Eubacterium]*	0.20 [0.09–0.31]	0.21 [0.17–0.30]	0.18 [0.10–0.50]	0.913
*Fusobacteria*			2.59 [1.68–3.34]	5.07 [2.06–6.78]	5.22 [4.34–8.00]	0.074
	*Uncl*. *Fusobacteriaceae*	*Uncl*. *Fusobacteriaceae*	1.80 [1.06–2.65]	4.08 [1.91–5.40]	3.85 [3.25–5.72]	0.026*
	*Fusobacteriaceae*	*Uncl*. *Fusobacteriaceae*	0.66 [0.62–0.94]	0.99 [0.15–1.37]	1.50 [0.82–2.27]	0.063
*Proteobacteria*			6.60 [4.69–6.72]	12.51 [10.62–13.91]	11.64 [9.42–16.49]	0.011*
*Betaproteobacteria (class)*			0.01 [0.01–0.03]	0.19 [0.10–0.32]	0.12 [0.04–0.16]	0.002**†
	*Burkholderiales (order)*		0.74 [0.58–0.98]	2.53 [1.29–2.95]	2.04 [0.59–2.62]	0.055
	*Alcaligenaceae*	*Sutterella*	2.19 [1.61–2.69]	3.31 [2.53–4.91]	3.79 [1.72–7.07]	0.348
	*Methylophilaceae*	*Methylotenera*	0.00 [0.0–0.0]	0.00 [0.0–0.0]	0.00 [0.0–0.0]	1.000
	*Desulfovibrionaceae*	*Uncl*. *Desulfovibrionaceae*	0.15 [0.13–0.21]	0.30 [0.19–0.43]	0.23 [0.12–0.32]	0.122
		*Desulfovibrio*	0.89 [0.22–1.68]	1.14 [0.65–1.55]	1.31 [0.73–1.44]	0.840
	*Campylobacteraceae*	*Campylobacter*	1.70 [1.13–2.07]	4.92 [1.45–6.64]	4.02 [2.37–6.68]	0.080
	*Succinivibrionaceae*	*Uncl*. *Succinivibrionaceae*	0.03 [0.01–0.05]	0.16 [0.14-.18]	0.30 [0.14–0.57]	0.007**†
		*Succinivibrio*	0.37 [0.19–0.80]	0.24 [0.11–0.28]	0.16 [0.08–0.36]	0.234
	*Enterobacteriaceae*	*Uncl*. *Enterobacteriaceae*	0.02 [0.0–0.04]	0.01 [0.0–0.09]	0.04 [0.02–0.08]	0.252
	*Pseudomonadaceae*	*Pseudomonas*	0.00 [0.0–0.0]	0.00 [0.0–0.0]	0.00 [0.0–0.0]	1.000

*p<0.05 and

**p<0.01.

†Benjamini-Hochberg corrected p-value <0.15.

**Fig 6:**
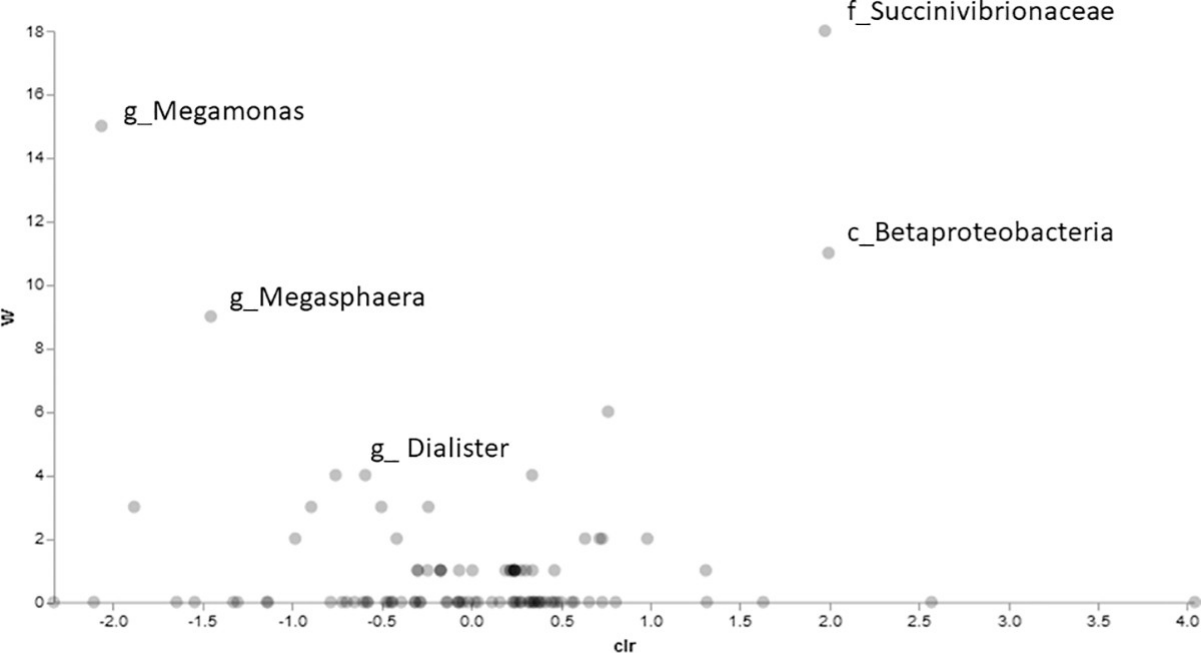
Differentially abundant microbial taxa identified by Analysis of Composition of Microbiomes (ANCOM). Volcano plot of differential abundance comparing kittens prior to versus after experimental infection with *T*. *foetus*. Clr (x-axis) is a measure of the effect size difference for a particular species between the two conditions. The W-statistic (y-axis) is the strength of the ANCOM test for the tested number of species.

**Fig 7:**
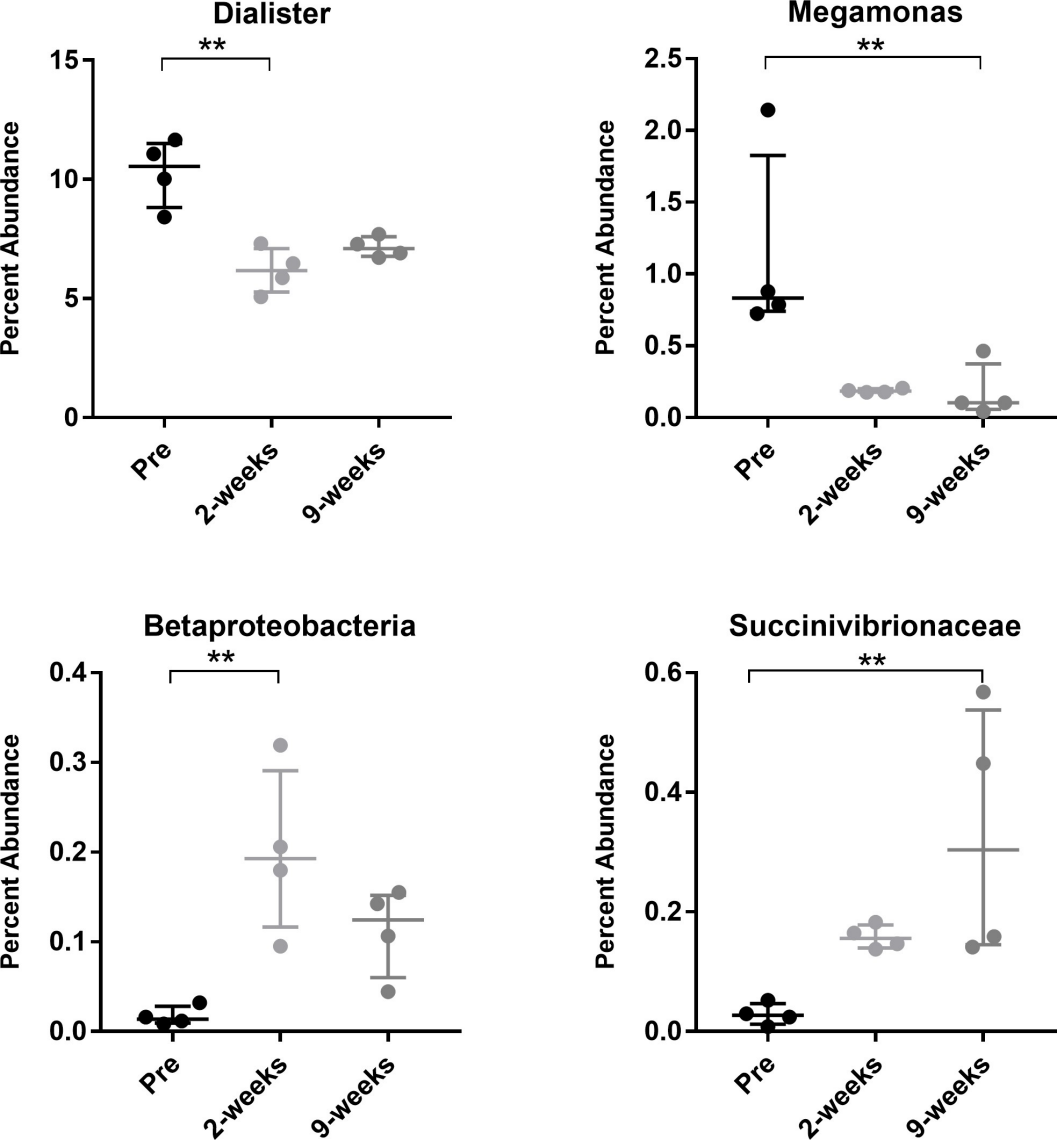
Percent abundance of *Dialister*, *Megamonas*, *Betaproteobacteria*, and *Succinivibrionaceae* in feces of 4 kittens prior to (Pre) and at 2 time points after (2 weeks and 9 weeks) experimental infection with feline *T*. *foetus*. Data points represent individual cats. Bars represent median and interquartile range. **p<0.01 (Benjamini-Hochberg-corrected p<0.15).

After accounting for the impact of multiple statistical comparisons, four taxa were identified as significantly impacted by introduction of *T*. *foetus* infection. Following experimental infection with *T*. *foetus*, the fecal microbiota of kittens had a significantly lower abundance of members of the genus *Dialister* and *Megamonas* and significantly greater abundance of members of the class *Betaproteobacteria* and family *Succinivibrionaceae* compared to kittens prior to experimental infection (Figs 6 and 7).

## Discussion

To better understand the role of the colonic microbiota in the pathogenesis of *T*. *foetus* infection in cats, we characterized the fecal microbiota of 89 cats undergoing testing for natural infection. Each fecal sample was submitted for diagnostic *T*. *foetus* PCR testing based on clinical signs such as diarrhea, history of exposure, or for purposes of screening for the infection. Overall, 19% of cats in this population were diagnosed with *T*. *foetus* while the other 81% had any number of other potential causes for their clinical signs. Based on 16S rRNA gene sequencing, *T*. *foetus* infection did not alter the predominant bacterial phyla present in feces which were similar in identity to prior descriptions of the fecal microbiota in cats [34–38]. These phyla were represented mainly by Firmicutes, Bacteroidetes, Proteobacteria, Actinobacteria, and Fusobacteria. The presence of *T*. *foetus* also did not affect the overall number or diversity of different taxa present in the microbial community. However, *T*. *foetus* had a significant impact on community membership that could be attributed to increases in both the presence and relative abundance of the genus *Megamonas* (phylum Firmicutes, family Veillonellaceae) and *Helicobacter* (phylum Proteobacteria). A key attribute of this study was our ability to extend this population level observation to assessment of the specific impact of experimentally introduced *T*. *foetus* infection in a group of purpose-bred kittens that were controlled for age, sex, diet, and background microbiome. In these kittens, introduction of *T*. *foetus* resulted in a decrease in relative abundance of the genus *Megamonas* and *Dialister* (phylum Firmicutes, family Veillonellaceae) and significantly increased abundance of members of the class *Betaproteobacteria* and family *Succinivibrionaceae* (phylum Proteobacteria).

It is remarkable that changes in the family Viellonellaceae and the specific genus *Megamonas* were specifically and significantly impacted in both naturally occurring and experimentally induced *T*. *foetus* infection. Both *Megamonas* and *Dialister* produce large amounts of short chain fatty acids such as propionate and lactate which are thought to possess anti-inflammatory properties and provide a large portion of the energy supply for colonocytes [39]. A higher abundance of *Megamonas* in the microbial community is a distinguishing characteristic of healthy cats [40]. Decreases in these taxa, as observed in kittens with experimentally induced *T*. *foetus* infection, is commonly associated with disease states such as IBD [40–42]. Low levels of short chain fatty acids may lead to colonocyte autophagy which could contribute to the pathogenic effects of *T*. *foetus* or somehow benefit its survival.

It is likely that *T*. *foetus* promotes dysbiosis in part by competition for fermentable substrates. In mice, the presence of fermentable fiber is a requirement for successful intestinal colonization with the commensal trichomonad *Tritrichomonas musculis* [43]. Specific colonic bacterial species also compete for this fiber source. Studies have identified that the availability of fermentable fiber can affect the abundance of *Megamonas* and other members of the family *Veillonellaceae* [44–48]. For example, cats fed a chicken-based extruded diet had agreater abundance of *Megamonas* species compared to those fed raw whole chicks [45]. Similarly, administration of fermentable prebiotics containing fructooligosaccharides or inulin results in a higher abundance of *Veillonellaceae* in the feces of cats [46] and specifically the genus *Megamonas* in dogs [47]. By consuming available fiber, *T*. *foetus* may indirectly decrease the abundance of *Megamonas* and *Dialister* via competition for available nutrients.

The major fermentation products of *T*. *foetus* are succinate, acetate, and molecular hydrogen [49]. Succinate is also a key substrate or product of metabolism by the *Veillonellaceae* (*Megamonas* and *Dialister*) and *Succinovibrionaceae* [50]. Succinate is an important pro-inflammatory signaling molecule [50] that may be of interest as a mediator of disease pathogenesis in *T*. *foetus* infection. In mice, colonization with *T*. *musculis* increases the concentration of intestinal succinate which stimulates tuft cell succinate receptors leading to activation of a type 2 immune response [43, 51]. Activation of the host epithelial inflammasome by *T*. *musculis* exacerbates the development of T- cell- driven colitis [52]. Succinate increases in the intestinal lumen of mice and humans with inflammatory bowel disease and is correlated with disease activity [53]. A decrease in succinate-consuming bacterial strains is reported in people with IBD [54]. Accordingly, it is worth considering that metabolic products of *T*. *foetus* or the dysbiotic microbiota contribute to the pathogenesis of colonic inflammation and diarrhea in cats with *T*. *foetus* infection. If this is the case, then manipulation of dietary fermentation substrates, addition of competing probiotics, or administration of select metabolites might influence *T*. *foetus* survival, secondary microbial dysbiosis, or pathogenic effects.

In contrast to experimentally infected kittens, cats with naturally occurring *T*. *foetus* infection had a significant increase in abundance of *Megamonas* as well as *Helicobacter*. This is an interesting finding as a recent study comparing the microbiome of normal cats versus those with chronic and acute diarrhea found a significant increase in *Helicobacter* and *Megamonas* in the normal cats as compared to those with diarrhea [40]. This finding in our naturally infected cats could indicate resolving dysbiosis with a more chronic *T*. *foetus* infection as compared to the acute stages of experimental infection. Other explanations could include use of a single strain of *T*. *foetus* for the experimental infection that resulted in a unique effect, differences in age between the two populations of cats, underlying differences in microbiome ecology between the purpose-bred kittens and an outbred population of cats, or the use of different DNA extraction methods for naturally infected cats vs. experimentally infected kittens. It is also possible that cats with a higher abundance of *Megamonas* or *Helicobacter* are more susceptible to infection by *T*. *foetus* via changes to the colonic microenvironment. A similar phenomenon has been described in women where changes in the abundance and specific identity of vaginal *Lactobacillus* species are linked to increased risk for development of *T*. *vaginalis* infection [22].

Inherent in the outbred population of cats included in this study were numerous variables having potential impact on the microbiota including differences in age, breed, sex, clinical signs of diarrhea, and treatments administered [34, 37, 40, 55, 56]. Additional influences that were unknown for these cats include differences in diet, environment, comorbidities, or co-infections [35, 36, 38, 56–58]. While these variables have a demonstrated impact on the fecal microbiota in studies controlling for their effects, their impact in this large, cross-sectional study population was not found to be significant. We did however observe a significant impact of fecal collection method on composition of the microbiome. These results likely reflect preferential sampling of microbiota from different microbial niches. In the present study, *Prevotella* was observed in greater abundance in fecal samples collected with a loop than by the other methods. In people, *Prevotella* is a predominant taxa in the rectum where it is observed in higher abundance in samples of the rectal mucosa compared to feces [59]. Samples collected with a loop were more likely to disrupt the mucosal surface microbiota thereby increasing recovery of *Prevotella*. A significantly lower abundance of *Campylobacter* was observed in samples collected using the flush method in our study. In pigs, *Campylobacter* is significantly more abundant in the mucosa than in lumen content [60]. Accordingly, samples collected by flush were more likely to capture non-mucosa-associated bacteria. These observations emphasize both the impact of sample collection method on results of microbiome analysis as well as support the power of our study to detect expected differences between these methods. All cats in the study had a treatment history provided and those reporting use of antimicrobials were excluded. Reports of other treatments were too few in number to enable a robust examination of treatment effects on microbiota composition.

In this first study of the fecal microbiota in cats with and without *T*. *foetus* infection, specific changes in the abundance of members of the *Veillonellaceae* and *Succinivibrionaceae* suggest altered fermentative metabolism in the colon of cats with *T*. *foetus* infection. These findings support additional investigations into the composition and functional impacts of the fecal metabolome on colonic inflammation and diarrhea in cats with *T*. *foetus* infection.
